# Pre- and Post-debridement Wound Cultures in Gustilo Type III Open Tibial Fractures to Predict Wound Infection at a Tertiary Care Hospital

**DOI:** 10.7759/cureus.64293

**Published:** 2024-07-10

**Authors:** Md. Towhidul Islam, Md. Zahidur Rahman, Md. Syedur Rahaman, Tapas Mandal, Shekhar K Bosu, Md. Emdadul H Bhuiyan

**Affiliations:** 1 Department of Orthopaedic Surgery, National Institute of Traumatology and Orthopaedlc Rehabilitation (NITOR), Dhaka, BGD; 2 Department of Orthopaedics and Traumatology, National Institute of Traumatology and Orthopaedic Rehabilitation (NITOR), Dhaka, BGD

**Keywords:** open tibia fracture, open tibial fracture, wound culture, resistance pattern, debridement, infection

## Abstract

Background: Gustilo type III open tibial fractures are difficult injuries that carry a higher risk of infection and other consequences. Open-fracture wound microbiology is dynamic and responsible for change over time. Effective antibiotic treatment plans are required, as detrimental microorganisms are often linked to these types of lesions.

Objectives: The study aimed to determine whether pre- and post-debridement wound cultures could predict wound infection in Gustilo type III open tibial fractures.

Methods: This prospective study was carried out at the National Institute of Traumatology and Orthopaedic Rehabilitation (NITOR) in Dhaka, Bangladesh, on 344 patients who presented to the emergency department with a Gustilo type III open tibial fracture within 24 hours of injury from June 2018 to October 2019. Three successive cultures were carried out: one in the emergency room (surveillance culture), the second at the emergency theater after debridement, and the third in the ward after one week (seven to 10 days). Statistical analyses of the results were conducted using Microsoft Excel (Microsoft Corp., Redmond, WA) and IBM SPSS Statistics for Windows, version 27 (IBM Corp., Armonk, NY).

Results: The study included 344 patients with an average age of 37.15 years, with motor vehicle accidents being the primary cause (78.2%). Gustilo type IIIA fractures made up the majority (48.5%), followed by type IIIB fractures (44.8%). A significant reduction in contamination rates was observed from initial admission (48.8%) to post-debridement (36.6%) (p =.001). There was a significant positive correlation between pre-debridement cultures and wound infections (r =.311), as well as between post-debridement cultures and wound infections. The infection rate increased to 61.6% in ward samples, indicating a high rate of hospital-acquired infections. *Pseudomonas *and *Klebsiella *species were the most prevalent multidrug-resistant bacteria that caused these infections.

Conclusion: The present study provides information on the relationship between contamination and infection. Gram-negative pathogens were dominant in this study, and the results of the antibiograms showed an alarming pattern of resistance. Nosocomial infection demands further urgent study.

## Introduction

The Gustilo and Anderson classification system, which was subsequently modified by Gustilo et al., is widely used to grade open fractures [1, 2]. In this system, type I indicates a puncture wound of ≤1 cm with minimal contamination or muscle crushing. Type II open fractures are characterized by a laceration of more than 1 cm in length, accompanied by moderate soft-tissue damage and crushing. Importantly, bone coverage remains adequate, and comminution (fragmentation) is minimal. Type III open fractures are further subclassified into types A, B, and C according to the severity of the soft tissue injury, the need for vascular reconstruction, and the worsening prognosis. A type IIIA open fracture entails significant soft-tissue damage, typically caused by a high-energy injury with severe crushing. However, there is adequate soft-tissue coverage over the bone. Type IIIB signifies extensive soft tissue damage, often with periosteal stripping and bone exposure, accompanied by severe contamination and bone fragmentation. Flap coverage is necessary to provide sufficient soft-tissue coverage. A type IIIC fracture involves an arterial injury requiring repair, indicating a severe vascular component to the injury.

Open fractures typically result from high-energy injuries, often involving significant force. In these injuries, not only is the bone fractured, but there is also exposure of the bone and deep tissue to the external environment [2]. This leads to contamination as well as the introduction of foreign bodies and microbes into the wound [1]. The mechanism of injury (road traffic accident, fall from a height, gunshot injury) has a significant impact on the microbiological pattern at the wound site. In many cases, it has been observed that infections in open fractures are often nosocomial in origin. Because the offending organisms are quite different in the ward sample from the initial sample during admission, [3] a prospective cohort study from Serbia on 277 patients found the highest prevalence rates of nosocomial infections in the orthopedic wards [4]. Malunion, nonunion, chronic osteomyelitis, and sometimes even amputation might be the ultimate consequences of this infection [1, 5].

The management protocol for open fractures typically involves several steps. It begins with initial resuscitation, followed by patient assessment and classification of the injury. Antibiotic therapy is initiated, and then debridement and wound management are performed. Fracture stabilization is crucial, often followed by early bone grafting and supplementary procedures aimed at promoting healing [1, 6]. Appropriate surgical debridement is considered the most important procedure for open lower limb fracture management. Irrigation of the open wound is an essential and very commonly performed orthopedic procedure. A prospective, randomized study at Columbia concluded that irrigation of open fracture wounds with an antibiotic solution offers no advantages over the use of a nonsterile soap solution [7].

The principles of judicious antibiotic use and guidelines for infection control have been extensively disseminated through publications. However, adherence to these recommendations is often lacking [8]. The World Health Organization (WHO) has warned that antibiotic resistance poses a major global threat and a post-antibiotic era in which common infections and even minor injuries can threaten life [9]. Irrational use of antibiotics has resulted in the development of a progressively antibiotic-resistant microbial ecosystem worldwide, including Bangladesh [10].

Several investigations have proposed that organisms identified in wounds before debridement closely resemble those causing subsequent infections [11]. Conversely, contrasting findings indicate no significant association, suggesting infections primarily stem from nosocomial organisms [12]. Understanding these bacterial patterns is crucial for formulating antibiotic protocols for prophylaxis and empirical treatment. However, local studies examining these patterns are currently lacking. This study aimed to describe the pathogens of pre- and post-debridement bacterial cultures, antibiotic susceptibility, and their relation to wound infection.

## Materials and methods

From January 2018 to December 2019, a cross-sectional study was conducted in the Department of Orthopedics, National Institute of Traumatology and Orthopaedic Rehabilitation (NITOR), Dhaka, Bangladesh. In all, 344 patients took part in the research purposefully. After obtaining consent and ensuring eligibility criteria alignment, patient data regarding the variables of interest were gathered using a pre-established structured questionnaire through interviews and observations. A sample was selected through a convenience non-probability sampling method from patients who attended the emergency department of NITOR with a Gustilo type III open fracture of the tibia within 24 hours of injury.

During initial resuscitation in the emergency room, a surveillance culture sample (from the wound before the administration of the prophylactic antibiotic) was collected and sent. Prophylactic antibiotics (intravenous flucloxacillin and third-generation cephalosporin) were administered. Then, patients were sent to the emergency theater for wound debridement, fracture stabilization, and soft tissue care. Debridement was done following current practice at NITOR using chlorhexidine (Hexiscrub), normal saline, hydrogen peroxide, and povidone-iodine solution. A second post-debridement culture (the last saline wash from the wound at the emergency theater) was sent. From the theater, after initial fracture stabilization, stable patients were sent to the postoperative ward, followed by the general ward. A third infection culture sample was sent after admission to the ward at seven to 10 days for culture sensitivity and identification of the organism. A questionnaire was filled out by the investigator, containing information regarding demographic variables, mechanism and time of injury, time of wound debridement (time elapsed since injury in hours), Gustilo type III fracture characteristic, and the results of three successive culture sensitivity tests.

Data processing and analysis

The collected data were edited for entry into Microsoft Excel (Microsoft Corp., Redmond, WA). Analysis was done on IBM SPSS Statistics for Windows, version 27 (IBM Corp., Armonk, NY). The data were tabulated, and quantitative parameters such as the age of the patient were summarized in terms of mean with standard deviation and percentage. The results were analyzed using Chi-square tests to see the difference between the two variables. The Pearson correlation test was used to see the correlation among variables. The significance of the results was determined using a 95.0% confidence interval, with a value of p <0.05 considered to be statistically significant.

## Results

**Figure 1:**
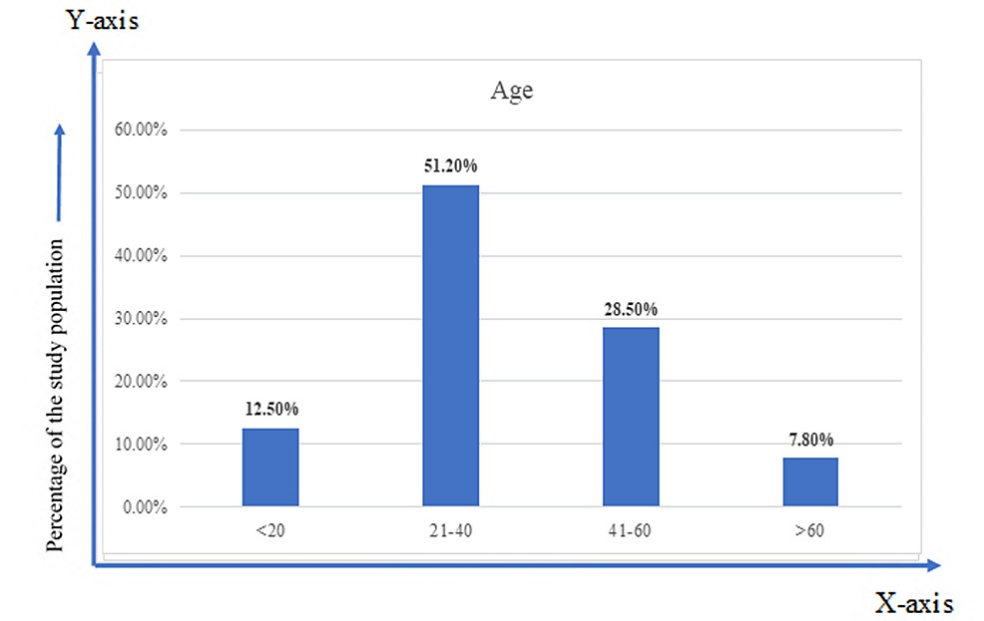
Age-wise distribution of the study population (n=344)

The mean±SD ages of the patients were 37.15±15.40 years, with motor vehicle accidents being the predominant cause for the fracture (78.2%). The most common fractures (48.5%) were of Gustillo type IIIA, followed by Gustillo type IIIB (44.8%). There was a significant difference in the contamination rate between the on-admission culture and the post-debridement culture, with the contamination rate declining from 48.8% to 36.6% after debridement. The mean age of the patients was 37.15 years; 51% of patients were in the age group of 21-40 years (Figure 1).

**Figure 2:**
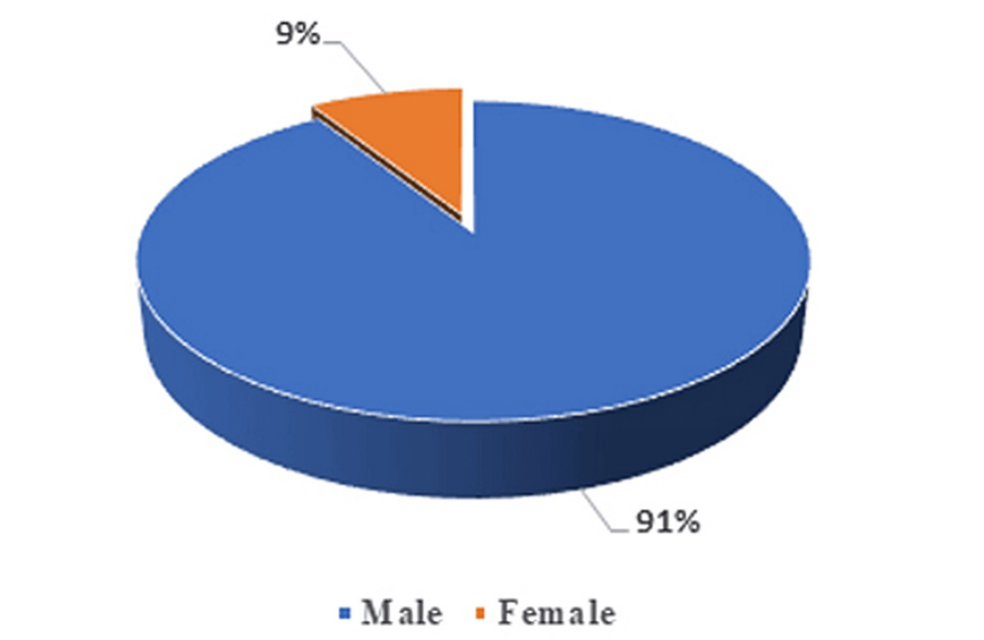
Gender-wise distribution of the study population (n=344)

The pie chart in Figure 2 shows the gender distribution of the study patients. The majority (91.3%) of the patients were male, and 8.7% were female.

**Figure 3:**
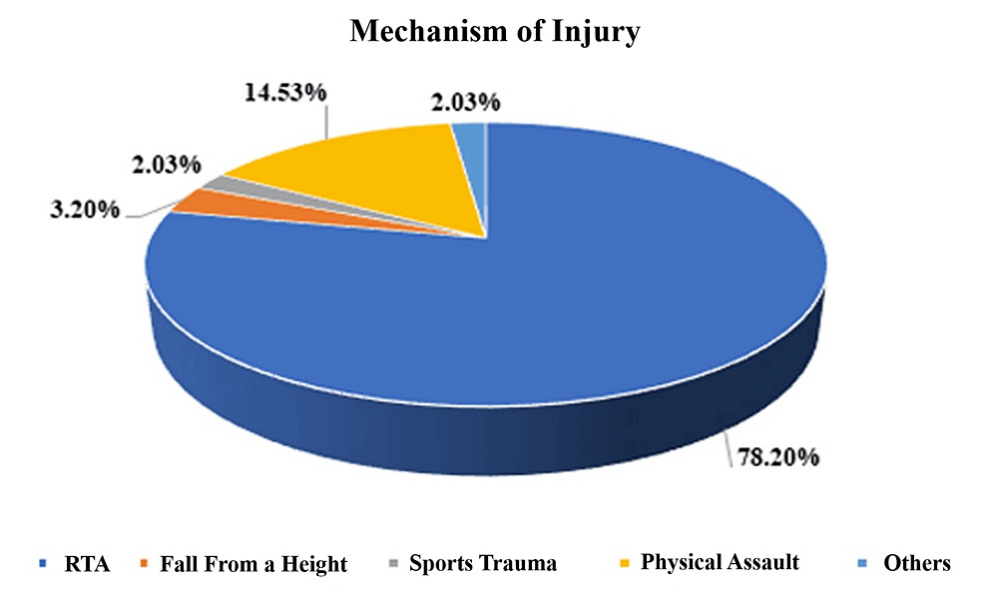
A pie diagram showing the mechanism of injury (n = 344) RTA: road traffic accidents

The predominant mechanism of injury was road traffic accidents (78.20%), followed by physical assault (14.53%), falls from height (3.20%), sports trauma (2.03%), and others (Figure 3).

**Figure 4:**
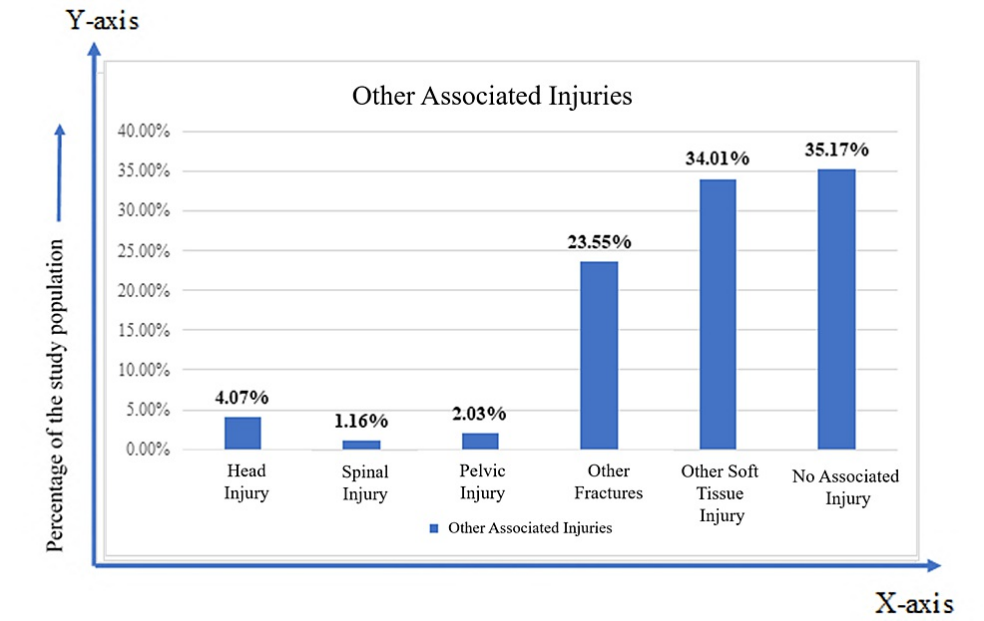
Bar diagram showing other associated injuries (n = 344)

There was no associated injury in 35.17% of cases. Among the associated injuries, concomitant soft tissue injuries (34.01%) were the most common, followed by various types of fractures (23.55%) (Figure 4).

Gustilo type III subclassification and Gustilo type IIIA fracture were predominant in 167 (48.5%) cases, followed by type IIIB, in 154 (44.8%) cases. The highest infection/contamination rate was in Gustilo type IIIB (136, 52.50%) cases, followed by type IIIA (105, 40.54%) cases (Table 1).

**Table 1:** Injury characteristics and overall infection/contamination rate shown according to Gustilo III subclassification (subtypes) (n = 344).

Type of fracture	Injury characteristics n (%)	Overall infection/contamination rate n (%)
Gustilo IIIA	167(48.5)	105 (40.54)
Gustilo IIIB	154 (44.8)	136 (52.50)
Gustilo IIIC	23 (6.7)	18 (6.94)
Total	344 (100)	259 (100)

On admission, the contamination rate was 48.8%. Infection rates among the Gustilo and Anderson classifications, including subtypes, were as follows: type IIIB was predominant (131, 37.1%), followed by IIIA (104, 29.5%), and type II (86, 24.4%) (Table 2).

**Table 2:** Contamination present in pre-debridement culture and post-debridement culture (n = 344).

Infection	Pre-debridement culture n (%)	Post-debridement culture n (%)
Present	168 (48.8)	126 (36.6)
Absent	176 (51.2)	218 (63.4)
Total	344 (100)	344 (100)

A positive surveillance culture was noted in 168 (48.4%) patients at admission. The contamination rate decreased to 126 (36.6%) after debridement. After debridement, only 42 (12.2%) patients were contamination-free. Following debridement, only 42 patients (12.2%) remained free from contamination (Table 3).

**Table 3:** Details of participants who remained contamination-free after debridement

Culture	Contamination	Frequency n (%)	Contamination-free after debridement n (%)
On admission (surveillance culture/first culture positive)	Present	168 (48.8)	168-126 = 42 (12.2)
	Absent	176 (51.2)	
After debridement (post-debridement culture/second culture positive)	Present	126 (36.6)	
	Absent	218 (63.4)	

The infection present in the ward samples again rose to 61.6% from post-debridement contamination of 36.6%, which indicated hospital-acquired infection. The highest infection rate was in Gustilo type IIIB, 117 (55.1%), followed by type IIIA, 90 (42.8%) (Table 4).

**Table 4:** Infection present in admitted cases (ward sample culture-positive) and infection rate shown according to the Gustilo III subclassification (subtypes) (n = 344)

	n (%)
Infection present	
Present	212 (61.6)
Absent	95 (27.6)
Healed	37 (10.8)
Total	344 (100)
Infection rate	
Gustilo IIIA	90 (42.8)
Gustilo IIIB	117 (55.1)
Gustilo IIIC	5(2.3)
Total	212 (100)

Table 5 demonstrates that 25% of non-contaminated cases become infected after admission.

**Table 5:** Contamination and infection were observed in the post-debridement culture and the third culture (culture from samples obtained in the ward).

Culture	Contamination	Frequency n (%)	Newly acquired infection after admission n (%)
Post-debridement contamination (positive second culture)	Present	126 (36.6)	212-126 = 86 (25%)
	Absent	218 (63.4)	
Infection from the ward sample (positive third culture)	Present	212 (61.6)	
	Absent and healed	132 (38.4)	

Table 6 demonstrates a significant positive correlation between the result of surveillance culture and wound infection (r=.311).

**Table 6:** Correlation between pre-debridement culture and wound infection

Correlations		Contamination present in pre-debridement culture	Infection present in the infection culture
Relation between pre-debridement culture and wound infection (n = 344)			
Contamination present in pre-debridement culture	Pearson correlation sig. (two-tailed) N	1 344	311** .000 .307
Infection present in infection culture	Pearson correlation sig. (two-tailed) N	.311** .000 .307	1 307
Relation between post-debridement culture and wound infection (n = 344)			
Contamination present in pre-debridement culture	Pearson correlation sig. (two-tailed) N	1 344	288** .000 .307
Infection present in infection culture	Pearson correlation sig. (two-tailed) N	.288** .000 .307	1 307

Various organisms were identified in three consecutive cultures. The count of organisms decreased in the second culture following debridement but rose again in the third culture (a sample taken from the ward). This indicates a hospital-acquired infection. Gram-negative organisms were predominant in all the cultures (Table 7).

**Table 7:** Common organisms found in three cultures (n = 344)

Bacteria	Fist culture		Second culture		Third culture	
	Freq.	%	Freq.	%	Freq.	%
Staphylococcus aureus	16	8.2%	5	3.4%	0	0%
*Streptococcus *sp.	4	2.07%	4	2.7%	8	3.3%
Escherichia coli	31	16.1%	19	12.9%	13	5.4%
*Pseudomonas *sp.	33	17.1%	39	26.5%	121	50.4%
*Klebsiella *sp.	44	22.7%	31	21.1%	68	28.3%
Citrobacter freundii	6	3.1%	4	2.7%	0	0%
*Proteus *sp.	9	4.6%	11	7.4%	15	6.2%
Acinetobacter	34	17.6%	18	12.2%	9	3.7%
*Serratia *sp.	2	1.0%	0	0%	0	0%
Providencia alcalifaciens	0	0%	0	1.6%	1	0.4%
*Enterobacter *sp.	13	6.7%	12	8.1%	3	1.2%
Flavobacterium	1	0.5%	0	0%	0	0%
*Plesiomonas *sp.	0	0%	2	1.3%	0	0%
Aeromonas	0	0%	2	1.3%	0	0%
Morganellamoganii	0	0%	0	0%	2	0.8%
Total	193	100%	147	100	240	100

## Discussion

The incidence of complications in Gustilo type III open fractures is affected by several factors, including the presence of soft-tissue defects, contamination of wounds during the injury, and extended stays in the hospital that increase the risk of exposure to surgical pathogens. The emergence of multidrug-resistant organisms has made management further complicated. Despite encouraging developments in the management of open fractures, the infection rate in type IIIB open fractures, especially in the tibia, is still a major problem. This study conducted an assessment of 344 instances of open tibial fractures treated at the emergency department of a tertiary care orthopedic hospital in Bangladesh. The aim was to analyze the bacterial cultures obtained before and after debridement, along with antibiotic susceptibility, and their correlation with eventual wound infections.

The mean age of the patients in this study was 37.15 ± 15.40 years. The mean age in a nine-year research on Gustilo type III tibial fractures was found to be 38 years [13]. Bangladesh noticed a mean age of 34 years in an investigation on open tibial fractures at NITOR [14]. A different study by the Royal College of England found a mean age of 37 years [15]. All of these research findings correspond with our own. In our analysis, there were 314 (91%) male patients and 30 (9%) female patients, indicating a male predominance. Male predominance was also seen in several other studies [16].

In 78.20% of cases, automobile accidents were the most frequent mechanism of injury. Road traffic accidents are the most frequent cause of fractures (64.7%), according to a new study on Gustilo type III open tibial fractures [17]. On the other hand, Finland found that road traffic accidents were the second most common injury mechanism, accounting for only 15% of all injuries that occur in younger age groups. In this study, among the associated injuries, soft tissue injuries were the most common (34.01%), followed by fractures (23.55%) and head injuries (4.07%). A large-scale study in the USA found head injuries were the most commonly associated injuries [18]. One important reason behind this difference might be that their study was in a first-world setting. 

Regarding the degree of damage based on the Gustilo subclassification, type IIIA fractures accounted for the majority (48.5%), with type IIIB fractures coming in second (44.8%). Type IIIB was determined to be the highest subtype in a review article that included 32 eligible publications with 3,036 tibia patients; nevertheless, type IIIA is the subtype that we studied [19]. This difference is not very significant because, in our study, the difference between Gustilo types IIIA and IIIB was only 3.7%. In this study, the infection rate of Gustilo type IIIB was highest (55.18%), followed by type IIIA (42.85%).

From the results of the pre-debridement culture (first culture) and the post-debridement culture, it is evident that some patients were contamination-free after debridement (42, 12.2%). However, when we considered the result of the third culture from the ward sample, the infection rate rose to 61.6% from the post-debridement contamination of 36.6%, which indicates that 25% of non-contaminated cases became infected after admission. This is commonly known as a nosocomial infection. The findings of a prospective study conducted over a one-and-a-half-year period at a tertiary care hospital in India revealed that nosocomial infections are a major global issue, with rates that vary from as low as 1% in a few European and American countries to more than 40% in several Asian countries [17]. This range matches the results of our research. 

In regards to qualitative cultures' potential to identify infection, 84% of positive pre-debridement cultures result in infection in the end. Thus, of the 69% of patients who became infected, 60% had a positive culture at the time of pre-debridement, and 40% had a negative culture.

This explains why pre-debridement cultures are 60% sensitive. Similarly, infection resulted in 87% of positive post-debridement cultures. Therefore, of the 69% of patients that were infected, only 55% had negative debridement cultures, and 45% had cultures that were positive at the time of debridement. Consequently, debridement cultures had a 45% sensitivity and an 84% specificity.

This assertion is supported by a study done at St. John's Medical College Hospital in Bangalore, India, which found that post-debridement cultures have higher specificity and pre-debridement cultures have higher sensitivity for infection detection [11]. The present investigation's conclusions show a relationship between early wound contamination and infection. Significant positive correlations between wound infection, surveillance, and post-debridement cultures were discovered in this investigation. In accordance with the results of another Indian investigation, cultures obtained during debridement were more sensitive in estimating the infection rate than pre-debridement or surveillance cultures [4].

A bacteriological study found that the common organisms present in our study in three cultures were *Escherichia coli* (*E. coli*), *Pseudomonas *sp., and *Klebsiella *sp. A cross-sectional study carried out in the department of microbiology at Chittagong Medical College, Bangladesh, found *E. coli *(39.77%), followed by *Klebsiella *species (22.73%) and *Pseudomonas *species (14.21%), which is almost similar to this study [20]. The multidrug resistance of common gram-positive and gram-negative organisms in our study's culture is troubling. While the majority of *Pseudomonas *species exhibit a sensitivity of approximately 55% to intravenous imipenem or meropenem, *Klebsiella *demonstrated the highest sensitivity of any medication used in this study, at 65%. The field of bacteriology provides a similar picture of nosocomial infection.

This study found that pre- and post-debridement wound culture in the Gustilo type III open tibial fracture has a significant role in predicting wound infection. Still, hospital-acquired infections are common in orthopedically admitted patients. Further, a multicenter, randomized, large sample study is required to differentiate the strain pattern of the same bacteria found in three cultures, which would better explain the nosocomial infection.

Limitations of the study

Due to expenditure and planning constraints, the current study was completed in a very short amount of time. Another drawback of the current investigation was the tiny sample size.

## Conclusions

The field of diagnostic microbiology is essential to the management of infection. Infection in open fractures can be found using pre- and post-debridement cultures. In this investigation, gram-negative organisms predominated, and antibiogram results revealed a concerning pattern of resistance. More urgent research on nosocomial infections is necessary. This study can act as a trial period for much larger studies, including numerous centers that will be able to validate the models of regression suggested here for use in the future, provide a picture of the country, and highlight areas that need to be improved in terms of management and adherence.
